# Impact of physical activity levels on the association between air pollution exposures and glycemic indicators in older individuals

**DOI:** 10.1186/s12940-024-01125-8

**Published:** 2024-10-18

**Authors:** Hyunji Park, Sun Young Kim, Heeseon Jang, Yae Won Ha, Young Mi Yun, Kwang Joon Kim, Yumie Rhee, Hyeon Chang Kim, Chang Oh Kim, Jaelim Cho

**Affiliations:** 1 Department of Public Health, Graduate School, Yonsei University, Seoul, Republic of Korea; 2 Department of Cancer Control and Population Health, Graduate School of Cancer Science and Policy, National Cancer Center, Goyang, Republic of Korea; 3 Department of Preventive Medicine, Yonsei University College of Medicine, 50-1 Yonsei-ro, Seodaemun-gu, Seoul, Republic of Korea; 4 Division of Geriatrics, Department of Internal Medicine, Yonsei University College of Medicine, Seoul, Republic of Korea; 5 Endocrine Research Institute, Department of Internal Medicine, Severance Hospital, Yonsei University College of Medicine, Seoul, Republic of Korea; 6 Institute for Environmental Research, Yonsei University College of Medicine, Seoul, Republic of Korea; 7 Institute of Human Complexity and Systems Science, Yonsei University, Incheon, Republic of Korea

**Keywords:** Air pollution, Glycemic indicators, Older individuals, Physical activity

## Abstract

**Background:**

Air pollution may exacerbate diabetes-related indicators; however, the longitudinal associations between air pollutant concentrations and glycemic markers remain unclear. In this prospective cohort study, we examined the longitudinal associations between air pollution and glycemic indicators among older individuals with normoglycemia at baseline and determined whether these associations differed according to changes in physical activity levels.

**Methods:**

Overall, 1,856 participants (mean age, 70.9 years) underwent baseline and 4-year follow-up surveys. We used linear mixed-effect models to examine the associations between previous 1-year exposures to air pollutants and glycemic indicators. We further investigated associations between previous 5-year exposures to air pollutants and glycemic indicators after the inverse probability of treatment weighting (IPTW). We explored effect modifications by the level of physical activity maintenance and changes in metabolic equivalent of task (METs) for physical activity.

**Results:**

Levels of particulate matter with aerodynamic diameters ≤ 10 μm (PM_10_) and ≤ 2.5 μm, and nitrogen dioxide (NO_2_) were significantly associated with increased fasting blood glucose, Hemoglobin A1c, insulin, and homeostatic model assessment for insulin resistance (HOMA-IR) values. After IPTW, the associations remained significant for PM_10_ and NO_2_. The positive associations of NO_2_ with insulin and HOMA-IR remained significant in the maintained inactive group, but not in the maintained moderate-to-vigorous active group. The positive associations of PM_10_ or NO_2_ with insulin and HOMA-IR remained significant in the group with increased METs, but not in those with decreased METs. In the post-hoc analysis of non-linear relationships between an increase in METs and glycemic indicators, insulin and HOMA-IR remarkably increased in the higher PM_10_ and NO_2_ exposure group from the point of 12,000 and 13,500 METs-min/week increase, respectively.

**Conclusions:**

We demonstrated longitudinal associations between air pollution exposures and increased insulin resistance in older individuals. Maintaining moderate-to-vigorous physical activity may mitigate the adverse effects of air pollution on insulin resistance. In older individuals dwelling in highly polluted areas, an increase of less than 12,000 METs-min/week may be beneficial for insulin resistance.

**Supplementary Information:**

The online version contains supplementary material available at 10.1186/s12940-024-01125-8.

## Highlights

**Why did we undertake this study?**

Air pollution exposures may increase the risk of diabetes mellitus; however, the impacts of changes in physical activity on the associations between air pollution exposures and glycemic indicators remained unclear.

**What is the specific question(s) we wanted to answer?**

We investigated the associations of previous 1-year and 5-year air pollution exposures with glycemic indicators in older individuals with normoglycemia and explored the potential impacts of changes in physical activity levels on the associations.

**What did we find?**

Previous 1-year exposures to PM_10_ and NO_2_ were associated with elevated fasting blood glucose, Hemoglobin A1c, insulin, and insulin resistance. Previous 5-year exposures to PM_10_ and NO_2_ were associated with increased insulin resistance. The adverse effects of NO_2_ existed in those who maintained inactive but not in those with maintained moderate-to-vigorous activity.

**What are the implications of our findings?**

Our findings underscore the detrimental impact of air pollution on glycemic health and emphasize that maintaining moderate to vigorous physical activity may mitigate the adverse effects of air pollution on insulin resistance in older individuals.

**Supplementary Information:**

The online version contains supplementary material available at 10.1186/s12940-024-01125-8.

## Background

Diabetes mellitus poses a huge global healthcare system burden. Environmental risk factors contribute 19.6% to the burden of diabetes mellitus [1]. Particularly, mounting evidence suggests that ambient air pollution is a major environmental risk factor for the development of diabetes mellitus. A meta-analysis of 21 cohort studies has shown that each 10-unit increase in particulate matter with aerodynamic diameters ≤ 10 μm (PM_10_) or ≤ 2.5 μm (PM_2.5_) was associated with an approximately 11% increase in the risk of incident diabetes [2]. Another meta-analysis (21 studies) demonstrated that each 10-unit increase in nitrogen dioxide (NO_2_) was associated with a 9% higher diabetes prevalence and a 4% increase in diabetes incidence, though the latter result was not statistically significant [3].

Existing evidence suggests that air pollution exposure may worsen the indicators related to diabetes mellitus [4–6]. Although numerous studies have demonstrated cross-sectional and longitudinal associations between air pollution and glycemic indicators (e.g., insulin resistance, fasting glucose, and hemoglobin A1c [HbA1c]) [4–6], little evidence exists on longitudinal associations in older individuals. To our knowledge, only one longitudinal study has found positive associations between 1-year average concentrations of PM_10_, PM_2.5_, and NO_2_ and changes in insulin resistance and insulin levels among older individuals [7].

Mounting evidence suggests that physical activity benefits glycemic control [8, 9]. However, there are mixed findings on the impact of physical activity on the relationship between air pollution and glycemic outcomes [10–13]. A retrospective cohort study conducted in the Republic of Korea has demonstrated that moderate-to-vigorous physical activity was associated with an approximately 9% lower risk of diabetes mellitus, regardless of PM_10_ and PM_2.5_ levels [10]. A prospective cohort study conducted in Taiwan has shown that a moderate-to-high physical activity group with low PM_2.5_ levels had a 64% lower risk of type 2 diabetes than that in an inactive group with high PM_2.5_ levels [11]. A cross-sectional study conducted in China found that the beneficial effects of physical activity on diabetes mellitus outweighed the harmful PM_2.5_ effects in individuals with lower PM_10_ levels (< 92 µg/m^3^) and physical activity [12]. A UK Biobank study has reported no differences in the association of PM_10_, PM_2.5_, and NO_2_ concentrations with the risk of diabetes mellitus according to physical activity levels [13]. In the above longitudinal study by Zhang et al., there was no effect modification of PM_2.5_ and NO_2_ on glycemic status and insulin resistance by physical activity levels [7]. It is still unclear whether changes in the level of physical activity modify the association between air pollution exposure, glycemic status, and insulin resistance in individuals with normoglycemia. Further, it is worth investigating whether the potential beneficial effects of physical activity on glycemic outcomes can offset the adverse effects of air pollution.

Hence, this study explored the longitudinal associations between previous 1-year air pollution exposures and glycemic indicators among older individuals with baseline normoglycemia. Additionally, we investigated the associations between previous 5-year exposures to air pollution and 4-year glycemic indicator changes among the same population after the inverse probability of treatment weighting (IPTW) to improve causal inference. Subsequently, we examined whether these associations differed according to changes in physical activity levels.

## Methods

### Study cohort

The Korean Urban Rural Elderly (KURE) study aimed to identify ways to prevent and effectively treat important chronic diseases among the older individuals in the Republic of Korea [14]. Through local advertisements, we recruited participants aged 65 years and older from northwest Seoul (urban areas: Eunpyung-gu, Mapo-gu, and Seodaemun-gu) and Incheon (rural area: Ganghwa). At baseline, the participants completed questionnaires (demographics, history of disease, and lifestyle behaviors), underwent anthropometric measurements (height, weight, and blood pressure), and blood tests. The follow-up survey was conducted between 2016 and 2019 at a 4-year interval from the baseline between 2012 and 2015. Exclusion criteria were (1) a self-reported history of diabetes mellitus, fasting blood glucose ≥ 126 mg/dL, or HbA1c ≥ 6.5% (48 mmol/mol) at baseline, (2) failure to undergo a follow-up survey, (3) unidentifiable residential address, and (4) missing values for glycemic indicators or covariates. This cohort study was approved by the Institutional Review Board of Yonsei University Health System, Severance Hospital (IRB No. 4-2012-0172/4-2022-0435) and adhered to the principles of the Declaration of Helsinki. We obtained informed consent for participation from all patients.

### Air pollutants

The annual average concentrations of PM_10_, PM_2.5_, and NO_2_ were estimated at the residential addresses of participants using a validated exposure prediction model applied in previous cohort studies [15–17]. This nationwide prediction model was built in a universal kriging framework based on air quality regulatory monitoring data along with geographic predictors and spatial correlation. Geographic predictors were estimated by partial least squares from 320 geographic variables, including transportation, demographics, land cover, transportation facilities, emissions, greenness, and elevation. Model performance (cross-validation R^2^) for PM_10_, PM_2.5_, and NO_2_ in 2016 was 0.50, 0.37, and 0.81, respectively. We estimated air pollution concentrations for 1 year before the baseline and follow-up survey years (e.g., 2011 air pollution data used for the survey year of 2012). We also estimated concentrations for 5 years prior to the baseline survey years (e.g., 2011 − 2015 air pollution data used for the survey year of 2016). Because national air quality monitoring for PM_2.5_ began in 2015, PM_2.5_ concentrations in each survey year between 2012 and 2016 were replaced with 1-year concentrations in 2015.

### Glycemic indicators

Glycemic indicators included fasting blood glucose, HbA1c, insulin, and homeostatic model assessment for insulin resistance (HOMA-IR) at baseline and follow-up. HOMA-IR was calculated using the equation: $\:\left[Insulin\:\right(\mu\:U/mL)\:\times\:\:Fasting\:glucose\:(mg/dL\left)\right]/405$ [18].

### Physical activity

The level of physical activity was calculated based on the metabolic equivalent of task (METs) and categorized as inactive, minimally active, and health-enhancing physical activity (HEPA) [19]. The HEPA group consisted of participants who engaged in vigorous activity of over 1,500 METs-min/week for at least 3 days or involved in any combination of walking, moderate-intensity, and vigorous activity for at least 3,000 METs-min/week for at least 7 days. The minimally active group included individuals engaging in at least 20 min of vigorous activity per day for 3 or more days, or at least 30 min of moderate-intensity activity or walking per day for 5 or more days, or involved in any combination of walking, moderate-intensity, and vigorous activity for at least 600 METs-min/week for 5 or more days. The remaining participants were considered inactive group.

Changes in physical activity levels were determined using baseline and follow-up data and were categorized in terms of (1) levels of physical activity maintenance and (2) the change in METs. The levels of physical activity maintenance consisted of the inactivity and moderate-to-vigorous groups. Due to the small number of individuals in the maintained HEPA group, we combined the maintained minimally active group with the maintained HEPA group into the moderate-to-vigorous group. The change in METs was categorized as decreased (METs difference < 0) and increased METs (METs difference > 0); here, we excluded individuals whose METs did not change (METs difference = 0).

### Covariates

Demographics, socioeconomic factors, history of disease, lifestyle behaviors, blood pressure, and lipid profiles were considered covariates. Age (years), systolic blood pressure (SBP), diastolic blood pressure (DBP), as well as triglyceride, high-density lipoprotein (HDL) cholesterol, and low-density lipoprotein (LDL) cholesterol levels were included as continuous variables. LDL cholesterol levels were calculated using the Friedewald equation [20]. Categorical variables included sex (male or female), household income (quartile), physical activity (inactive, minimally active, or HEPA), smoking status (never, former, or current smoker), and current alcohol consumption status (none, monthly or less, or at least once a week).

### Statistical analysis

Linear mixed-effects models were used to examine the longitudinal associations of the previous 1-year exposure to air pollution with glycemic indicators, considering each participant as a random effect. All covariates except sex were considered as time-varying variables. Glycemic indicators, body mass index, and lipid profiles were natural log-transformed owing to a skewed distribution. Model 1 was adjusted for age and sex. Model 2 was adjusted for Model 1 variables plus household income, SBP, DBP, body mass index, triglycerides, HDL cholesterol, LDL cholesterol, level of physical activity, smoking status, and current alcohol consumption status. All air pollutant concentrations were standardized. The glycemic indicator change was expressed as a percentage change per 1-standard deviation (SD) increase in each air pollutant and its 95% confidence interval (CI). Percentage changes were calculated using the formula: $\:\left({exp}^{\beta\:}-1\right)\times\:100$. To perform a complete-case analysis, we excluded outcome variables, exposure data, and covariates from the analysis if any were missing. Selection of study participants is shown in Supplementary Material  1.

Additionally, we examined the associations of air pollution concentrations for 5 years prior to the baseline survey with glycemic indicators after IPTW [21]. This approach enabled us to improve causal inference by minimizing the impact of differences in baseline characteristics between participants living in high- and low-pollution areas. For IPTW, we estimated propensity scores by constructing multivariable logistic regression models, including all covariates except for physical activity. The propensity score was defined as the probability of being assigned to the higher or lower 5-year exposure group. The higher exposure group was participants with the 66 percentile or higher concentrations of PM_10_ (≥ 50.1 µg/m^3^), PM_2.5_ (≥ 23.9 µg/m^3^), and NO_2_ (≥ 32.5 ppb). The lower exposure group was those with lower than 33 percentiles of PM_10_ (< 47.3 µg/m^3^), PM_2.5_ (< 22.7 µg/m^3^), and NO_2_ (< 26.9 ppb). The numbers of these subsets were 633, 632, and 632 in the higher exposure groups and 612, 611, and 612 in the lower exposure groups for PM_10_, PM_2.5_, and NO_2_, respectively. Linear mixed-effects models were constructed by considering each matched pair as a random effect and simultaneously accounting for repeated measures within individuals. Furthermore, we included physical activity level as a time-varying covariate in the model.

Using the above lower and higher exposure subsets, we estimated the impact of physical activity level changes on the association between air pollution and glycemic indicators. We repeated the above linear mixed-effects model analyses with IPTW after stratification by changes in physical activity levels (levels of physical activity maintenance and the change in METs), including Model 2 covariates except for physical activity. Significant between-group differences were tested using the formula proposed by Altman and Bland [22].

Given the observed positive associations of PM_10_ and NO_2_ with insulin and HOMA-IR in the increased METs group, we conducted post-hoc analysis to explore non-linear relationships between the levels of METs increase and glycemic indicators at follow-up and compared the patterns between the lower and higher exposure groups. Participants with increased METs (METs difference between baseline and follow-up > 0) were only included in this post-hoc analysis. The non-linear relationships were estimated using a generalized additive model (GAM), including METs as a spline independent variable (degrees of freedom = 3) and each glycemic indicator as the dependent variable. The GAM was adjusted for the corresponding glycemic indicator at baseline and all covariates (as parametric variables) in Model 2 except physical activity.

All statistical analyses were performed using SAS version 9.4 (SAS Institute, Cary, NC, USA). Statistical significance was set at two-sided *p* < 0.05.

## Results

### Characteristics of the study cohort

We included 3,712 observations from 1,856 participants (585 men and 1,271 women; 1,603 urban and 253 rural dwellers) in the KURE cohort (Table 1). The baseline mean age (SD) was 70.9 (4.2) years. Participants were followed up for a mean of 4.0 (0.4) years. One-year mean (SD) concentrations of PM_10_, PM_2.5_, and NO_2_ at the baseline survey were 44.3 (4.1) µg/m^3^, 23.2 (1.2) µg/m^3^, and 25.3 (8.1) ppb, respectively. Five-year mean (SD) concentrations of PM_10_, PM_2.5_, and NO_2_ at the baseline survey by region were as follows: 49.0 (3.4) µg/m^3^, 23.2 (1.2) µg/m^3^, 30.7 (4.9) ppb for urban area, 47.4 (2.8) µg/m^3^, 23.5 (0.9) µg/m^3^, 7.2 (1.8) ppb for rural area. The baseline median (25%–75%) values of fasting blood glucose, HbA1c, insulin, and HOMA-IR levels were 92.0 (87.0–98.0) mg/dL, 5.6 (3.8–8.6) %, 5.6 (5.4–5.8) µU/mL, and 1.3 (0.9–2.0), respectively. An assessment of the balance of participant characteristics before and after IPTW by air pollutants is shown in Supplementary Material  2.

**Table 1:** Characteristics of the study cohort

		Baseline (*n* = 1856)	1st follow-up (*n* = 1856)
**Previous 1-year exposure**			
	PM_10_, µg/m^3^	44.3 ± 4.1	45.1 ± 3.8
	PM_2.5_, µg/m^3^	23.2 ± 1.2	24.6 ± 2.3
	NO_2_, ppb	25.3 ± 8.1	23.2 ± 7.6
**Previous 5-year exposure**			
	PM_10_, µg/m^3^	48.8 ± 3.4	45.7 ± 2.3
	PM_2.5_, µg/m^3^	23.2 ± 1.2	24.0 ± 1.1
	NO_2_, ppb	27.5 ± 9.3	24.5 ± 7.9
**Glycemic indicators**,** median (25%–75%)**			
	Fasting blood glucose (mg/dL)	92.0 (87.0–98.0)	91.0 (86.0–99.0)
	HbA1c (%)	5.6 (3.8–8.6)	5.0 (3.0–8.0)
	Insulin (µU/mL)	5.6 (5.4–5.8)	5.7 (5.5–5.9)
	HOMA-IR	1.3 (0.9–2.0)	1.2 (0.7–1.9)
**Age**,** years**		70.9 ± 4.2	74.8 ± 4.3
**Sex**,** N (%)**			
	Male	585 (31.5)	585 (31.5)
	Female	1271 (68.5)	1271 (68.5)
**Household income**,** N (%)**			
	Quartile 1 (≤$1,621)	437 (23.5)	396 (21.3)
	Quartile 2 ($1,622–$3,213)	472 (25.4)	500 (26.9)
	Quartile 3 ($3,214–$6,317)	497 (26.8)	498 (26.8)
	Quartile 4 (≥$6,318)	450 (24.2)	462 (24.9)
**Smoking status**,** N (%)**			
	Current smokers	95 (5.1)	70 (3.8)
	Former smokers	348 (18.8)	385 (20.7)
	Never smokers	1413 (76.1)	1401 (75.5)
**Current drinking status**,** N (%)**			
	None	1130 (60.9)	1146 (61.7)
	Monthly or less	374 (20.2)	384 (20.7)
	At least once a week	352 (19.0)	326 (17.6)
**Physical activity**,** N (%)**			
	Inactive	1221 (65.8)	386 (20.8)
	Minimally active	383 (20.6)	999 (53.8)
	HEPA	352 (19.0)	326 (17.6)
**Body mass index, median (25%–75%)**,** kg/m**^**2**^		24.0 (22.2–25.9)	24.3 (22.4–26.3)
**Systolic blood pressure**,** mmHg**		128.1 ± 15.2	132.4 ± 16.6
**Diastolic blood pressure**,** mmHg**		73.9 ± 8.7	73.4 ± 8.9
**Lipid profile**,** median (25%–75%)**			
	Triglycerides, mg/dL	112.0 (84.0–152.5)	113.0 (85.0–146.0)
	HDL cholesterol, mg/dL	50.0 (43.0–59.0)	53.0 (45.0–62.0)
	LDL cholesterol, mg/dL	106.2 (86.6–127.2)	102.6 (82.0–126.2)

*Abbreviations.* PM_10_: particulate matter with aerodynamic diameters ≤ 10 μm; PM_2.5_: particulate matter with aerodynamic diameters ≤ 2.5 μm; NO_2_: nitrogen dioxide; HbA1c: hemoglobin A1c; HOMA-IR: homeostatic model assessment for insulin resistance; HEPA: health-enhancing physical activity; HDL: high-density lipoprotein; LDL: low-density lipoprotein

*Footnote*: Values are expressed as mean (standard deviation) unless otherwise stated

### Longitudinal associations of previous 1-year air pollution exposures with glycemic indicators

In Model 2, a 1-SD increase in PM_10_ was associated with increased fasting blood glucose (percentage change: 0.93%, 95% CI: 0.61–1.24%, *p* < 0.001), HbA1c (0.84%, 0.67–1.02%, *p* < 0.001), insulin (4.42%, 2.91–5.94%, *p* < 0.001), and HOMA-IR (5.38%, 3.72–7.06%, *p* < 0.001) (Table 2). A 1-SD increase in PM_2.5_ was associated with increased HbA1c (0.68%, 0.49–0.87%, *p* < 0.001). A 1-SD increase in NO_2_ was associated with increased fasting blood glucose (1.15%, 0.72–1.58%, *p* < 0.001), HbA1c (0.38%, 0.10–0.66%, *p* = 0.009), insulin (8.70%, 6.66–10.77%, *p* < 0.001), and HOMA-IR (9.92%, 7.68–12.20%, *p* < 0.001).

**Table 2:** Longitudinal associations of previous 1-year air pollution exposures with glycemic indicators

Exposures		Model 1		Model 2	
		Percent changes (95% CI)	*p*-value	Percent changes (95% CI)	*p*-value
**PM**_**10**_					
	Fasting blood glucose, mg/dL	1.31 (1.02, 1.60)	< 0.001	0.93 (0.61, 1.24)	< 0.001
	HbA1c, %	1.11 (0.95, 1.27)	< 0.001	0.84 (0.67, 1.02)	< 0.001
	Insulin, µU/mL	5.09 (3.64, 6.56)	< 0.001	4.42 (2.91, 5.94)	< 0.001
	HOMA-IR	6.50 (4.90, 8.12)	< 0.001	5.38 (3.72, 7.06)	< 0.001
**PM**_**2.5**_					
	Fasting blood glucose, mg/dL	0.15 (-0.17, 0.47)	0.359	-0.22 (-0.55, 0.10)	0.173
	HbA1c, %	0.96 (0.78, 1.14)	< 0.001	0.68 (0.49, 0.87)	< 0.001
	Insulin, µU/mL	0.61 (-0.95, 2.19)	0.446	-0.53 (-2.02, 0.98)	0.488
	HOMA-IR	0.78 (-0.92, 2.52)	0.369	-0.75 (-2.37, 0.89)	0.368
**NO**_**2**_					
	Fasting blood glucose, mg/dL	1.22 (0.80, 1.65)	< 0.001	1.15 (0.72, 1.58)	< 0.001
	HbA1c, %	0.45 (0.16, 0.73)	0.002	0.38 (0.10, 0.66)	0.009
	Insulin, µU/mL	10.07 (7.64, 12.56)	< 0.001	8.70 (6.66, 10.77)	< 0.001
	HOMA-IR	11.45 (8.78, 14.18)	< 0.001	9.92 (7.68, 12.20)	< 0.001

***Footnotes***: Percent changes per 1-SD increment were from linear mixed regression models. Glycemic indicators, body mass index, and lipid profile (triglycerides, high-density cholesterol, Low-density cholesterol) were natural log-transformed due to skewed distribution. Model 1: adjusted for age and sex. Model 2: adjusted for Model 1 variables plus household income, systolic blood pressure, diastolic blood pressure, body mass index, triglycerides, high-density lipoprotein cholesterol, low-density lipoprotein cholesterol, level of physical activity, smoking status, and current alcohol consumption status

***Abbreviations***: CI: confidence interval; PM_10_: particulate matter with aerodynamic diameters ≤ 10 μm; PM_2.5_: particulate matter with aerodynamic diameters ≤ 2.5 μm; NO_2_: nitrogen dioxide; HbA1c: hemoglobin A1c; HOMA-IR: homeostatic model assessment for insulin resistance

### Associations of previous 5-year air pollution exposures with glycemic indicators after IPTW

Compared with the lower PM_10_ exposure group, the higher PM_10_ exposure group had significantly increased insulin (7.74%, 1.51–14.35%, *p* = 0.014) and HOMA-IR (8.94%, 2.19–16.14%, *p* = 0.009) (Table 3). The higher NO_2_ exposure group had significantly increased fasting blood glucose (1.48%, 0.38–2.59%, *p* = 0.009), insulin (15.42%, 8.87–22.36%, *p* < 0.001), and HOMA-IR (17.19%, 10.01–24.83%, *p* < 0.001) compared with the lower NO_2_ exposure group. None of the associations of PM_2.5_ with any glycemic indicators was statistically significant.

**Table 3:** Associations of previous 5-year air pollution exposures with changes in glycemic indicators after the inverse probability of treatment weighting

Glycemic indicators		Number of the lower/higher exposure groups	Percent changes (95% CI)	*p*-value
**PM**_**10**_		612/633 (1,244/1,247 after IPTW)		
	Fasting blood glucose, mg/dL		1.10 (-0.01, 2.21)	0.051
	HbA1c, %		0.31 (-0.46, 1.08)	0.433
	Insulin, µU/mL		7.74 (1.51, 14.35)	0.014
	HOMA-IR		8.94 (2.19, 16.14)	0.009
**PM**_**2.5**_		611/632 (1,243/1,243 after IPTW)		
	Fasting blood glucose, mg/dL		0.16 (-0.91, 1.24)	0.769
	HbA1c, %		-0.04 (-0.81, 0.74)	0.921
	Insulin, µU/mL		-2.94 (-8.42, 2.88)	0.316
	HOMA-IR		-2.78 (-8.67, 3.50)	0.378
**NO**_**2**_		612/632 (1,246/1,241 after IPTW)		
	Fasting blood glucose, mg/dL		1.48 (0.38, 2.59)	0.009
	HbA1c, %		0.63 (-0.14, 1.42)	0.111
	Insulin, µU/mL		15.42 (8.87, 22.36)	< 0.001
	HOMA-IR		17.19 (10.01, 24.83)	< 0.001

***Footnotes***: Percent changes were from linear mixed regression models after the inverse probability of treatment weighting. Glycemic indicators were natural log-transformed due to skewed distribution. The model was adjusted for the level of physical activity

***Abbreviations***: CI: confidence interval; PM_10_: particulate matter with aerodynamic diameters ≤ 10 μm; PM_2.5_: particulate matter with aerodynamic diameters ≤ 2.5 μm; NO_2_: nitrogen dioxide; HbA1c: hemoglobin A1c; HOMA-IR: homeostatic model assessment for insulin resistance; IPTW: inverse probability of treatment weighting

### Associations of previous 5-year air pollution exposures with glycemic indicators, stratified by changes in physical activity levels

After stratification by level of physical activity maintenance, NO_2_ was significantly associated with increased insulin levels in the maintained inactive group (30.30%, 13.62–49.43%, *p* < 0.001) (Table 4). This association did not remain significant in the maintained moderate-to-vigorous group (-6.01%, -21.47–12.50%, *p* = 0.500) (interaction *p* = 0.005). Additionally, NO_2_ was significantly associated with increased HOMA-IR in the maintained inactive group (33.70%, 15.34–54.98%, *p* < 0.001). This association did not remain significant in the maintained moderate-to-vigorous active group (-6.83%, -23.07–12.85%, *p* = 0.470) (interaction *p* = 0.003). For PM_10_ and PM_2.5_, the association did not significantly differ between the maintained inactive group and the maintained moderate-to-vigorous group.

After stratification by changes in METs, NO_2_ was significantly associated with increased insulin (15.12%, 7.54–23.23%, *p* < 0.001) and HOMA-IR (16.77%, 8.50–25.67%, *p* < 0.001) in the increased METs group (Table 5). PM_10_ was significantly associated with increased insulin (7.59%, 0.20–15.53%, *p* = 0.044) and HOMA-IR (8.69%, 0.74–17.28%, *p* = 0.032) in the increased METs group.

**Table 4:** Associations of previous 5-year air pollution exposures with changes in glycemic indicators after the inverse probability of treatment weighting in the maintained physical activity groups

Exposures		Maintained inactive		Maintained moderate-to-vigorous activity		*p* for interaction
		Percent changes (95% CI)	*p*-value	Percent changes (95% CI)	*p*-value	
**PM**_**10**_	*Actual No. of the lower/higher exposure groups (post-IPTW No. of the lower/higher exposure groups)*	79/119 (196/200)		136/77 (211/223)		
	Fasting blood glucose, mg/dL	1.80 (-1.14, 4.83)	0.234	0.99 (-1.66, 3.71)	0.469	0.691
	HbA1c, %	-0.03 (-1.88, 1.86)	0.977	0.89 (-1.24, 3.06)	0.419	0.529
	Insulin, µU/ml	18.10 (2.65, 35.88)	0.021	-4.35 (-20.13, 14.56)	0.630	0.070
	HOMA-IR	20.18 (3.41, 39.66)	0.018	-3.37 (-20.14, 16.92)	0.725	0.078
**PM**_**2.5**_	*Actual No. of the lower/higher exposure groups (post-IPTW No. of the lower/higher exposure groups)*	178/35 (213/216)		86/120 (210/203)		
	Fasting blood glucose, mg/dL	2.62 (-0.40, 5.72)	0.092	0.41 (-2.06, 2.95)	0.745	0.274
	HbA1c, %	0.39 (-1.58, 2.40)	0.703	1.56 (-0.17, 3.32)	0.080	0.387
	Insulin, µU/ml	-8.83 (-20.98, 5.17)	0.206	1.28 (-12.92, 17.80)	0.869	0.321
	HOMA-IR	-6.52 (-19.94, 9.14)	0.394	1.70 (-13.55, 19.62)	0.839	0.462
**NO**_**2**_	*Actual No. of the lower/higher exposure groups (post-IPTW No. of the lower/higher exposure groups)*	84/102 (188/186)		147/40 (187/185)		
	Fasting blood glucose, mg/dL	2.67 (-0.23, 5.65)	0.073	-0.89 (-3.74, 2.05)	0.549	0.091
	HbA1c, %	0.01 (-1.91, 1.96)	0.995	-0.37 (-2.50, 1.81)	0.736	0.798
	Insulin, µU/ml	30.30 (13.62, 49.43)	< 0.001	-6.01 (-21.47, 12.50)	0.500	0.005
	HOMA-IR	33.70 (15.34, 54.98)	< 0.001	-6.83 (-23.07, 12.85)	0.470	0.003

Footnote: Percent changes were from linear mixed regression models after the inverse probability of treatment weighting. Glycemic indicators were natural log-transformed due to skewed distribution

Abbreviations: CI: confidence interval; PM_10_: particulate matter with aerodynamic diameters ≤ 10 μm; PM_2.5_: particulate matter with aerodynamic diameters ≤ 2.5 μm; NO_2_: nitrogen dioxide; IPTW: inverse probability of treatment weighting; HbA1c: hemoglobin A1c; HOMA-IR: homeostatic model assessment for insulin resistance

**Table 5:** Associations of previous 5-year air pollution exposures with changes in glycemic indicators after the inverse probability of treatment weighting in the decreased and increased physical activity groups

Exposures		Decreased physical activity		Increased physical activity		*p* for interaction
		Percent changes (95% CI)	*p*-value	Percent changes (95% CI)	*p*-value	
**PM**_**10**_	*Actual No. of the lower/higher exposure groups (post-IPTW No. of the lower/higher exposure groups)*	272/50 (321/329)		393/497 (890/891)		
	Fasting blood glucose, mg/dL	-0.80 (-3.23, 1.69)	0.523	1.01 (-0.32, 2.36)	0.138	0.206
	HbA1c, %	-1.13 (-2.89, 0.66)	0.216	0.44 (-0.50, 1.38)	0.361	0.129
	Insulin, µU/ml	-6.21 (-19.14, 8.79)	0.398	7.59 (0.20, 15.53)	0.044	0.102
	HOMA-IR	-6.91 (-20.59, 9.12)	0.378	8.69 (0.74, 17.28)	0.032	0.085
**PM**_**2.5**_	*Actual No. of the lower/higher exposure groups (post-IPTW No. of the lower/higher exposure groups)*	318/567 (886/885)		220/64 (285/270)		
	Fasting blood glucose, mg/dL	0.31 (-1.75, 2.41)	0.772	-0.04 (-1.33, 1.25)	0.946	0.777
	HbA1c, %	0.58 (-0.83, 2.01)	0.425	-0.33 (-1.25, 0.61)	0.492	0.296
	Insulin, µU/ml	-4.57 (-15.24, 7.44)	0.440	-3.59 (-9.97, 3.25)	0.296	0.883
	HOMA-IR	-4.27 (-15.70, 8.72)	0.502	-3.64 (-10.47, 3.71)	0.323	0.931
**NO**_**2**_	*Actual No. of the lower/higher exposure groups (post-IPTW No. of the lower/higher exposure groups)*	201/118 (318/323)		368/555 (927/920)		
	Fasting blood glucose, mg/dL	-0.48 (-2.89, 1.98)	0.697	1.40 (0.11, 2.70)	0.034	0.184
	HbA1c, %	-0.17 (-1.85, 1.54)	0.847	0.57 (-0.35, 1.50)	0.227	0.456
	Insulin, µU/ml	3.12 (-10.27, 18.51)	0.665	15.12 (7.54, 23.23)	< 0.001	0.164
	HOMA-IR	2.60 (-11.71, 19.22)	0.738	16.77 (8.50, 25.67)	< 0.001	0.129

Footnote: Percent changes were from linear mixed regression models after the inverse probability of treatment weighting. Glycemic indicators were natural log-transformed due to skewed distribution

Abbreviations: CI: confidence interval; PM_10_: particulate matter with aerodynamic diameters ≤ 10 μm; PM_2.5_: particulate matter with aerodynamic diameters ≤ 2.5 μm; NO2: nitrogen dioxide; IPTW: inverse probability of treatment weighting; HbA1c: hemoglobin A1c; HOMA-IR: homeostatic model assessment for insulin resistance

### Non-linear relationships between changes in physical activity levels and glycemic indicators in the higher and lower air pollution exposure groups

**Fig. 1:**
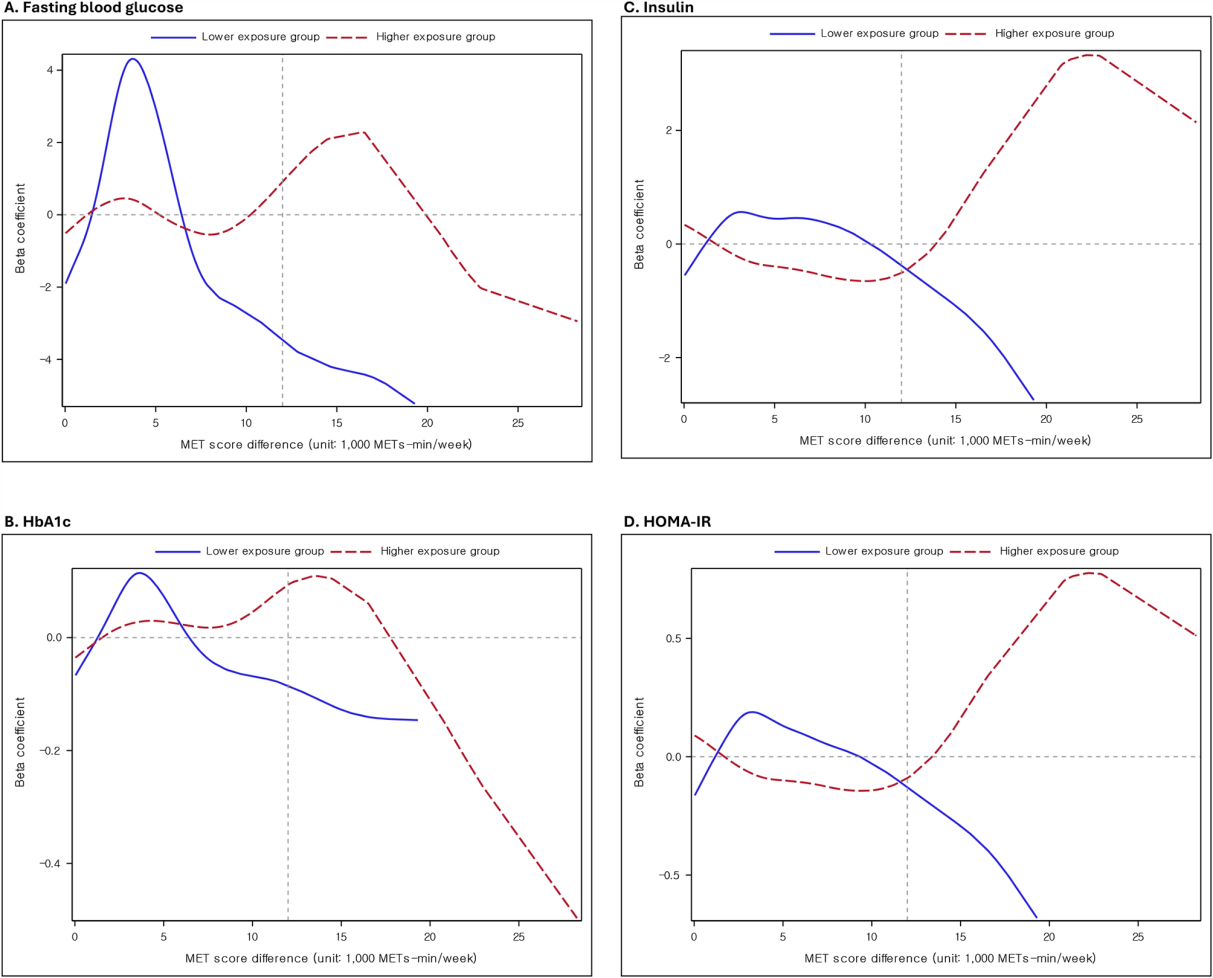
Non-linear relationships of changes in METs with (**A**) fasting blood glucose, (**B**) HbA1c, (**C**) insulin, and (**D**) HOMA-IR by the PM_10_ higher/lower exposure groups. ***Footnote***: Beta coefficients per 1,000 METs-min/week increment were from the generalized additive model, adjusting age, sex, household income, systolic blood pressure, diastolic blood pressure, body mass index, triglycerides, high-density lipoprotein cholesterol, low-density lipoprotein cholesterol, smoking status, and current alcohol consumption status. Glycemic indicators, body mass index, and lipid profile (triglycerides, high-density cholesterol, Low-density cholesterol) were natural log-transformed due to skewed distribution. ***Abbreviations***: METs; metabolic equivalent of task; HbA1c: hemoglobin A1c; HOMA-IR: homeostatic model assessment for insulin resistance; PM_10_; particulate matter with aerodynamic diameters ≤ 10 μm

PM_10_ concentrations ranged from 39.2 to 47.2 µg/m^3^ in the lower exposure group and from 50.1 to 61.3 µg/m^3^ in the higher exposure group. The direction of the associations of the level of METs increase with insulin, and HOMA-IR diverged from a 12,000 METs-min/week increase in both groups (Fig. 1). There were positive associations of the level of METs increase with insulin and HOMA-IR in the higher PM_10_ exposure group, but there were inverse associations in the lower PM_10_ exposure group from the point of approximately 12,000 METs-min/week increase.

**Fig. 2:**
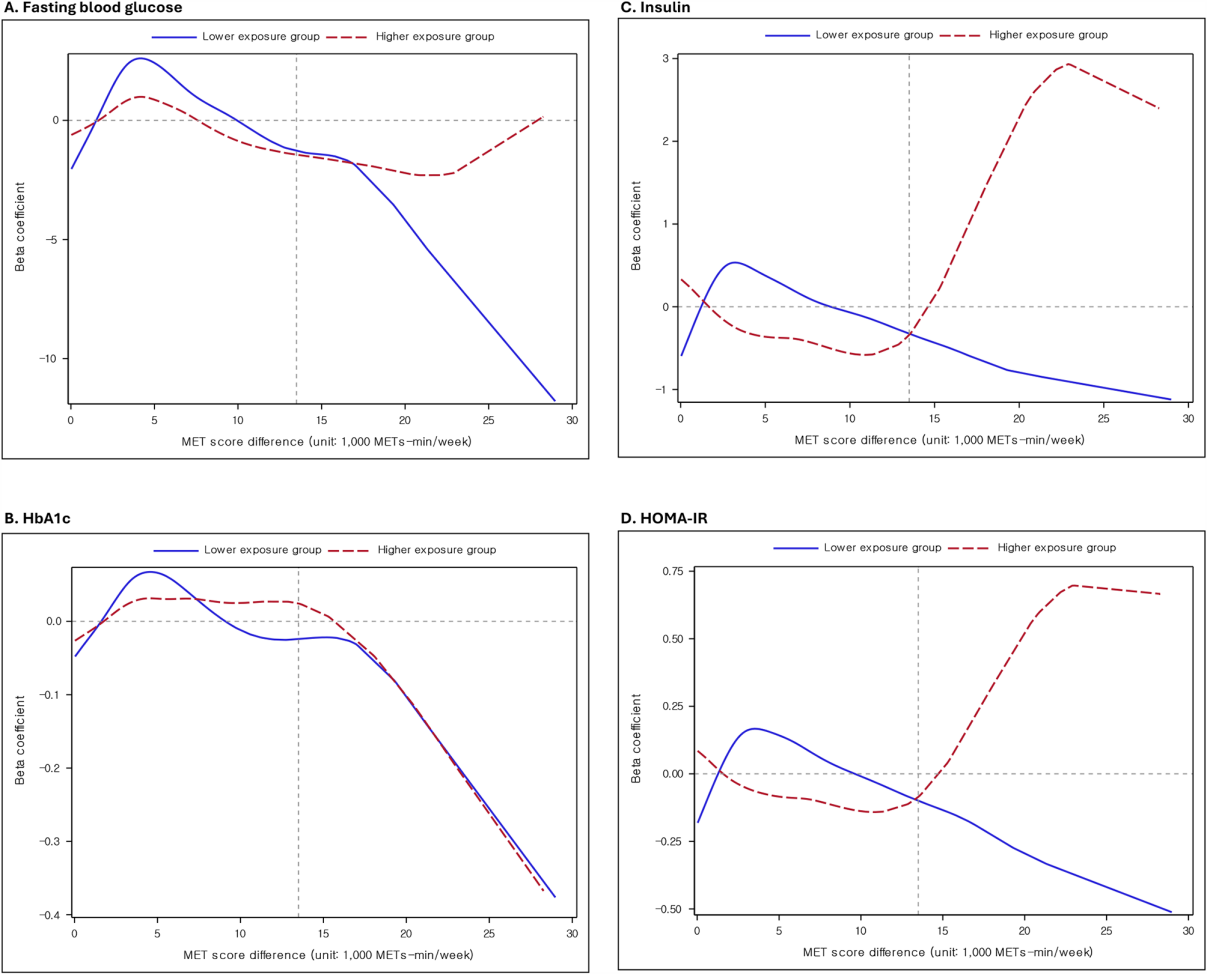
Non-linear relationships of changes in METs with (**A**) fasting blood glucose, (**B**) HbA1c, (**C**) insulin, and (**D**) HOMA-IR by the NO_2_ higher/lower exposure groups. ***Footnote***: Beta coefficients per 1,000 METs-min/week increment were from the generalized additive model, adjusting age, sex, household income, systolic blood pressure, diastolic blood pressure, body mass index, triglycerides, high-density lipoprotein cholesterol, low-density lipoprotein cholesterol, smoking status, and current alcohol consumption status. Glycemic indicators, body mass index, and lipid profile (triglycerides, high-density cholesterol, Low-density cholesterol) were natural log-transformed due to skewed distribution. ***Abbreviations***: METs; metabolic equivalent of task; HbA1c: hemoglobin A1c; HOMA-IR: homeostatic model assessment for insulin resistance; NO_2_: nitrogen dioxide

NO_2_ concentrations ranged from 5.7 to 26.8 ppb in the lower exposure group and 32.5 to 46.7 ppb in the higher exposure group. The direction of the associations of the level of METs increase with insulin, and HOMA-IR diverged from a 13,500 METs-min/week increase in both groups (Fig. 2). There were positive associations of the level of METs increase with insulin and HOMA-IR in the higher NO_2_ exposure group, but there were inverse associations in the lower NO_2_ exposure group from the point of approximately 13,500 METs-min/week increase. The non-linear relationships between the level of METs increase, and HbA1c did not remarkably differ between the lower and higher NO_2_ exposure groups.

## Discussion

This prospective cohort study investigated the longitudinal association between air pollution exposure and glycemic indicators in older individuals with normoglycemia. Previous 1-year exposures to PM_10_ and NO_2_ were associated with increased levels of fasting blood glucose, HbA1c, insulin, and HOMA-IR. Previous 5-year exposures to PM_10_ and NO_2_ were associated with increased insulin and HOMA-IR after applying IPTW to improve the causal inference. Changes in physical activity level modified these associations. Older individuals remaining in the inactive group consistently exhibited the adverse effects of PM_10_ and NO_2_ on insulin resistance; however, those remaining in the moderate-to-vigorous groups did not. Additionally, we found that the harmful effects of NO_2_ and PM_10_ on glycemic indicators existed in older individuals with increased physical activity levels but not in those with decreased physical activity. Further, an increase of less than 12,000 METs-min/week might be the optimal level to improve insulin resistance in older individuals dwelling in highly polluted areas.

This longitudinal study observed positive associations of PM_10_, PM_2.5_, and NO_2_ exposures with fasting blood glucose and HbA1c levels, similar to evidence from previous meta-analyses [5, 6]. We found longitudinal associations of PM_10_ and NO_2_ with increased fasting insulin and HOMA-IR levels among older individuals, which have only been reported in one previous study [7]. In this previous study, HOMA-IR increased by 3.1% for PM_2.5_ and 3.2% for NO_2_ per IQR increment and fasting insulin also exhibited similar associations (3.0% for PM_2.5_; 3.1% for NO_2_). Although there was no effect modification by baseline physical activity levels, adverse effects of coarse particles, PM_2.5_, and NO_2_ on HOMA-IR and fasting insulin tended to be stronger in the low level of physical activity group (versus those with medium or high levels of physical activity) [7]. In the present study, we evaluated physical activity levels both at baseline and 4-year follow-up and found effect modification by changes in physical activity levels. Specifically, we found that the adverse effects of NO_2_ on insulin resistance existed in older individuals who maintained inactive but not among those who remained in the moderate-to-vigorous active groups. These findings suggest that maintaining moderate-to-vigorous physical activity in older populations may mitigate the adverse effects of air pollution on glycemia and insulin resistance, consistent with findings of previous large-scale Asian studies on the association between air pollution and diabetes mellitus [10, 11].

Several mechanisms may underlie the effects of air pollution exposure on hyperglycemia and insulin resistance. Oxidative stress and systemic inflammation induced by air pollution exposure may disrupt glucose metabolism and increase the risk of developing diabetes mellitus [23–25]. Air pollution exposure may also activate the stress response of the endoplasmic reticulum, triggering inflammatory responses and abnormal insulin receptor substrate phosphorylation in the liver [26, 27]. Experimental evidence suggests that exposure to particulate matter may worsen insulin resistance via sympathetic nervous system activation, hypothalamic–pituitary–adrenal axis response excitement, and endothelial function changes [28–30]. While physical activity is protective against hyperglycemia by improving muscle insulin sensitivity, muscle capillary density, oxidative capacity, lipid metabolism, and insulin signaling pathways [9, 31, 32], the present study found that the adverse effects of PM_10_ or NO_2_ on insulin resistance existed in older individuals who increased their physical activity levels. A possible explanation is that increasing physical activity levels may reflect a longer duration of outdoor activities and/or higher respiratory rates during exercise, causing a higher internal dose of air pollution exposures. Alternatively, a certain level of physical activity might raise insulin resistance in highly polluted areas. In our post-hoc analysis to investigate this notion, insulin, and HOMA-IR drastically rose in the higher NO_2_ (≥ 32.5 ppb) exposure group from the point of 13,500 METs-min/week increase, and in the higher PM_10_ (≥ 50.1 µg/m3) exposure group from the point of 12,000 METs-min/week increase. By contrast, insulin and HOMA-IR decreased in the lower NO_2_ (5.7‒26.8 ppb) or PM_10_ (39.2–47.2 µg/m^3^) exposure group from these levels of METs-min/week increase. We propose that older individuals might improve insulin resistance by exercising even in highly polluted areas when they increase METs-min/week by less than 12,000 METs-min/week. An increase of 12,000 METs-min/week is also known to minimally reduce the risk of diabetes mellitus (by less than 1%), as demonstrated by a global meta-analysis [33].

There were several limitations to be noted in the present study. First, the characteristics of the study population may limit the generalization of the findings of this study. The KURE cohort was based on two geographical regions in the Republic of Korea, although we obtained sufficient spatial variability to efficiently detect the association between air pollution and glycemic indicators. In addition, the KURE cohort consisted of healthy older individuals. Participating in the study might have motivated the participants to have healthier lifestyle behaviors (low smoking and drinking rates and increasing physical activity), which may have underestimated the associations between air pollution exposures and glycemic indicators. Second, the response rate for the follow-up survey was 71.5% in the KURE cohort [14]. During the 4-year follow-up, 4.2% of the participants died, and 24.3% dropped out due to personal reasons such as immobility resulting from surgery or trauma, hospitalization, inability to communicate, relocation, etc. Compared with participants who completed follow-up, those who dropped out or died were older and had more underlying diseases such as hypertension, diabetes, and chronic kidney disease. Given that these participants may be more susceptible to the effects of air pollution exposures, the issue of drop-out may have led to conservative estimates. Third, the fit of our PM_2.5_ prediction model was relatively low, which may be attributable to the use of PM_2.5_ data from national air quality monitoring starting in 2015. This may explain the low variability of PM_2.5_ concentrations in our study and this might have led to the null associations between previous 5-year exposure to PM_2.5_ and glycemic indicators. Finally, there is a possibility of exposure misclassification. Our air pollution modeling was a residence-based ecological approach that could not consider individuals’ activity patterns. However, this limitation may not have distorted our results because we targeted older individuals who may have a small range of time-activity patterns [34].

## Conclusions

This prospective cohort study demonstrated longitudinal associations of previous 1-year exposures to PM_10_ and NO_2_ with increases in fasting blood glucose, HbA1c, and insulin resistance among older individuals without diabetes mellitus. We also found associations of previous 5-year exposures to PM_10_ and NO_2_ with insulin resistance after IPTW. We suggest that maintaining moderate-to-vigorous physical activity may mitigate adverse effects of air pollution exposures on insulin resistance. An increase of less than 12,000 METs-min/week might be the optimal level to improve insulin resistance in older individuals dwelling in highly polluted areas.

## Electronic supplementary material

Below is the link to the electronic supplementary material.

Supplementary Material 1

Supplementary Material 2

## Data Availability

No datasets were generated or analysed during the current study.

## Declarations

### Guarantor statement

COK and JC are the guarantors of this work and, as such, had full access to all the data in the study and take responsibility for the integrity of the data and the accuracy of the data analysis.

### Prior presentation

No part of the content in this manuscript has been presented or published in any form previously.

## Abbreviations

DBP: Diastolic blood pressure
GAM: Generalized additive model
HbA1c: Hemoglobin A1c
HEPA: Health-enhancing physical activity
HOMA-IR: Homeostatic model assessment for insulin resistance
IPTW: Inverse probability of treatment weighting
KURE: Korean Urban Rural Elderly
LDL: Low-density lipoprotein
METs: Metabolic equivalent of task
NO_2_: Nitrogen dioxide
PM_10_: Particulate matter with aerodynamic diameters ≤ 10 μm
PM_2.5_: Particulate matter with aerodynamic diameters ≤ 2.5 μm
SBP: Systolic blood pressure
HDL: High-density lipoprotein
SD: Standard deviation
