# Supplementary material for: Impact of physical activity levels on the association between air pollution exposures and glycemic indicators in older individuals
Source: Environ Health. 2024 Oct 18;23:87. doi: 10.1186/s12940-024-01125-8 (PMC11488365; doi:10.1186/s12940-024-01125-8)
Supplement: Supplementary file 1 — Supplementary Material 1 [file 12940_2024_1125_MOESM1_ESM.docx]

**Supplementary Material 1 N flow chart**
