# Supplementary material for: Impact of physical activity levels on the association between air pollution exposures and glycemic indicators in older individuals
Source: Environ Health. 2024 Oct 18;23:87. doi: 10.1186/s12940-024-01125-8 (PMC11488365; doi:10.1186/s12940-024-01125-8)
Supplement: Supplementary file 2 — Supplementary Material 2 [file 12940_2024_1125_MOESM2_ESM.docx]

**
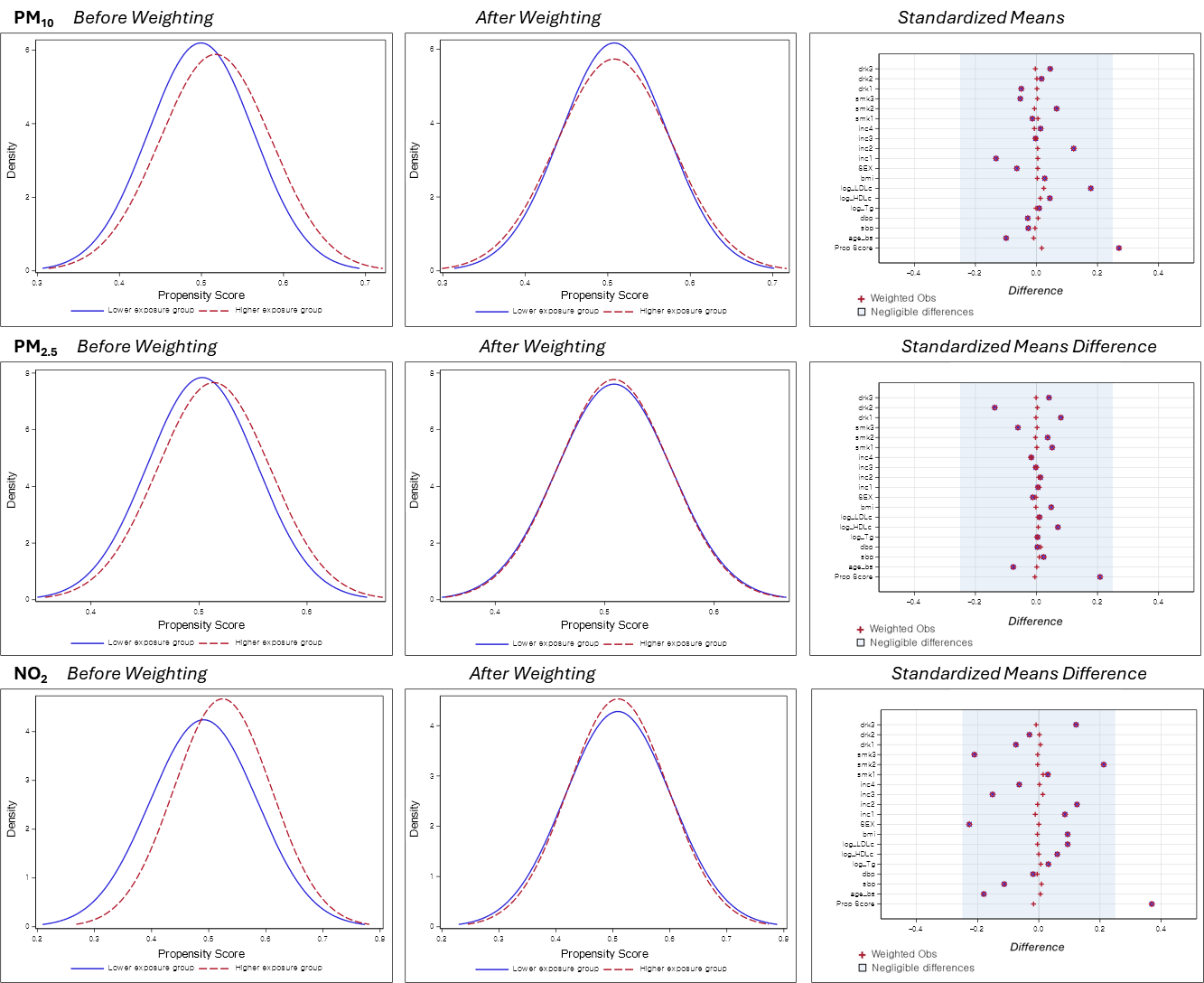
**

**Supplementary Material 2 Balance of participant characteristics before and after the inverse probability of treatment weighting by air pollutants.**

***Abbreviations***. PM_10_: particulate matter with aerodynamic diameters ≤10 μm; PM_2.5_: particulate matter with aerodynamic diameters ≤2.5 μm; NO_2_: nitrogen dioxide.
